# Heavy metal exposure, in combination with physical activity and aging, is related with oxidative stress in Japanese women from a rural agricultural community

**DOI:** 10.1186/s40064-016-2430-z

**Published:** 2016-06-24

**Authors:** Xiaoyi Cui, Mayumi Ohtsu, Nathan Mise, Akihiko Ikegami, Atsuko Mizuno, Takako Sakamoto, Masanori Ogawa, Munehito Machida, Fujio Kayama

**Affiliations:** Department of Environmental and Preventive Medicine, Jichi Medical University, 3311-1, Yakushiji, Shimotsuke, Tochigi 329-0498 Japan; Department of Pharmacology, Jichi Medical University, Shimotsuke, Tochigi Japan; Core Research for Evolutional Science and Technology, Japan Science and Technology Agency (CREST-JST), Tokyo, Japan

**Keywords:** Lead, Cadmium, Oxidative stress, Physical activity, Aging, Japanese female population

## Abstract

This study aimed to evaluate the relationships between oxidative stress and heavy metal exposure (lead [Pb] and cadmium [Cd]), as well as co-factors such as physical activity and age, in Japanese women. This study was conducted with female subjects from a rural agricultural community in Japan. Subjects were asked to complete lifestyle-related questionnaires and undergo a group health examination. Physical activity, alcohol consumption, body mass index, and other demographic information were collected. Blood and urine samples were collected to measure urinary 8-hydroxydeoxyguanosine (8-OHdG) levels and blood and urinary Cd and Pb concentrations. Urine samples were analyzed using high performance liquid chromatography and flameless atomic absorption spectrometry; blood samples were analyzed using inductively coupled plasma-mass spectrometry. Age, physical activity, and blood and urinary Cd and Pb concentrations were included in structural equation modeling analysis. Two latent factors for heavy metal exposure and physical activity were produced to predict the total influence of the variables. The final model was good: CMIN/DF = 0.775, CFI = 1.000, GFI = 0.975, AGFI = 0.954, RMSEA = 0.000. 8-OHdG levels were positively associated with heavy metal exposure, physical activity, and age (standard β of path analysis: 0.33, 0.38, and 0.20, respectively). Therefore, oxidative stress is associated with both, environmental and lifestyle factors, in combination with aging.

## Background

Oxidative stress can be defined as an excessive amount of reactive oxygen species (ROS), that overwhelm the innate antioxidant protection of the body and lead to oxidative damage to DNA, resulting in detrimental effects on health (Kohen and Nyska 2002; Poljsak et al. 2013). 8-hydroxy-2′-deoxyguanosine (8-OHdG), or 8-oxo-7,8-dihydro-2′-deoxyguanosine (8-oxodG), is considered to be a critical biomarker of oxidative stress and is a major product of oxidative DNA damage (Cheng et al. 1992; Valavanidis et al. 2009). Urinary 8-OHdG has been reported a good biomarker for and estimator of oxidative stress in humans after exposure to heavy metals (Pizzino et al. 2014; Valavanidis et al. 2009). In addition, urinary 8-OHdG could act as a prospective biomarker for the prediction of lifestyle-related diseases (Sakano et al. 2009).

A number of studies have investigated the effect of the heavy metals cadmium (Cd) and lead (Pb) on oxidative stress (Cuypers et al. 2010; Ercal et al. 2001; Flora et al. 2008; Mikhailova et al. 1997). In humans, Cd exposure occurs from cigarette smoking, air contamination, water, and food and has negative effects on human health owing to the low excretion rate and accumulation in the organs (Bertin and Averbeck 2006). The biologic half-life of Cd in the kidney may be up to 38 years (Agency for Toxic Substances and Disease Registry 2008). Previous studies with humans have also shown increased 8-OHdG concentrations associated with Cd exposure, with a strong association between urinary Cd and 8-OHdG (Engström et al. 2010). Pb is maintained in the environment; therefore, exposure to Pb can occur through various sources such as food, drinking water, and air contamination. No safe level of exposure to Pb has been identified. Various mechanisms have been found to explain Pb toxicity, and oxidative stress has been reported as a major mechanism of Pb-induced toxicity (Flora et al. 2012). The inhibitory effects of Pb on antioxidant enzymes render cells more susceptible to oxidative stress (Ercal et al. 2001).

A number of factors have been associated with oxidative stress. For example, oxidative stress increases during aging, and there is sufficient evidence that 8-OHdG might be a useful indicator of age-related oxidative stress (Mecocci et al. 1993; Wolf et al. 2005). In other studies, moderate exercise was associated with reduced 8-OHdG level, while smoking and long working hours were associated with increased 8-OHdG levels, suggesting that many lifestyle factors may affect oxidative stress (Irie et al. 2005; Kasai et al. 2001). We hypothesized that low and chronic exposure to the heavy metals Cd and Pb and the concurrent presence of lifestyle factors and older age are related with oxidative stress. Therefore, this study aimed to investigate the relationships between oxidative stress and heavy metal exposure as well as other co-factors and to determine if the associated risk factors could influence 8-OHdG concentrations, on an individual and/or concurrent basis.

## Methods

### Subjects

This study was conducted with female subjects from an agricultural community in a district in the north of Kyushu Island in 2001 as part of the Japanese Multi-centered Environmental Toxicant Study (JMETS). Additional details about the JMETS are reported elsewhere (Horiguchi et al. 2004a, 2005). The volunteer sampling method was used for the study. The subjects were asked to participate in the study through the local Agricultural Cooperative. The study area has a relatively low exposure to Cd and Pb, and the JMETS also demonstrated a relatively low exposure to Cd through rice. The subjects were farmers or family members of farmers who had consumed locally produced foods and rice and vegetables grown in their own fields since birth. Most of the residents had graduated high school, at most. The younger generation had completed higher education, such as junior college.

### Questionnaires and data collection

We held group orientations for the study subjects, during which we explained the study purpose and protocol and obtained written informed consent from each subject. The subjects completed the questionnaires after receiving an explanation from trained public health nurses. At the health examinations, we measured the subjects’ weight and height. Body mass index (BMI) was calculated using body weight and height.

Two questionnaires were completed. The first collected general information about socio-demographic characteristics, history of present or previous disease, smoking status, and self-reported amount of daily/weekly physical activity, categorized as voluntary exercise (consisting of moderate exercise, vigorous exercise, resistance training, and bicycle riding or walking), general everyday activities (consisting of meals, watching TV or reading, driving an automobile, and interesting activities at home), or activity at work (consisting of desk work, strenuous housework, active work, and carpentry work). We recorded the amount of weekly physical activity because many of the female subjects did not participate in voluntary exercise on a daily basis. The second questionnaire was a self-administered diet history questionnaire (DHQ) developed by Sasaki et al. (1998). Briefly, the DHQ was designed to determine food and nutrient intakes in the previous month in Japan. It has been validated and used in many epidemiological studies (Kobayashi et al. 2011). Alcohol consumption status was determined using the DHQ.

### Sample analysis

We collected peripheral blood and second-morning urine samples before breakfast. The whole blood samples were digested with nitric acid by a microwave device (MDS-200, CEM), after which blood Cd and Pb levels were measured using inductively coupled plasma-mass spectrometry (HP 4500 series; Yokokawa Analytical Systems, Tokyo, Japan). Urinary Cd and Pb levels were measured using flameless atomic absorption spectrometry (SIMAA 6000; Perkin Elmer, Japan). The urine samples were analyzed for 8-OHdG using the HPLC–CoulArray system (ESA Inc. USA). 8-OHdG stock standards were produced from 8-hydroxy-2′-deoxyguanosine (purity 99%, ESA, ECR703391). Urinary creatinine (Cr) was measured using the Jaffe reaction method (DIA-IATRON). The analytic value of urine samples was adjusted for Cr.

### Statistical analysis

Demographic variables were divided into groups based on age (<50, 50–59, and ≥60 years), BMI (<18.5, 18.5–24.9, and ≥ 25 kg/m^2^), smoking status (smokers and non-smokers), and alcohol consumption status (alcohol consumption, no alcohol consumption, previous alcohol consumption). Additionally, the 8-OHdG values were divided into four categories based on the 25th, 50th, and 75th percentiles. Non-parametric tests were performed for the variables that were not normally distributed over all groups or in each group (Shapiro–Wilk test, p < 0.05). The non-parametric Spearman’s correlation analysis was used to evaluate the relationships between 8-OHdG and the variables that were not distributed, including age, BMI, physical activity, and heavy metal exposure. The non-parametric Kruskal–Wallis test or Mann–Whitney test was performed to compare the median values of 8-OHdG between different demographic variable groups and median age and physical activity between different 8-OHdG categories. Heavy metal exposure variables were log-transformed to follow the approximate normal distribution to compare the geometric means (GMs). The GMs of heavy metal exposure and mean of BMI were compared between the 8-OHdG categories using the parametric analysis of variance (ANOVA) or Student’s *t* tests. To explore the causal relationship between heavy metal exposure, physical activity, age, and 8-OHdG, structural equation modeling (SEM) was used. We produced two latent factors for heavy metal exposure and physical activity to predict the total influence of the variables. All analyses were conducted using SPSS ver 19 (IBM Corp., Armonk, NY) and AMOS ver 20 (IBM Corp., Armonk, NY). A p < 0.05 was considered statistically significant.

## Results

After excluding 14 subjects (missing questionnaire data, n = 6; missing Cd concentration data for urine samples, n = 2; missing Cd concentration date for blood sample, n = 1; missing 8-OHdG concentration data for urine samples, n = 4; and colon cancer; n = 1) from the potential 202 subjects, the sample consisted of 188 subjects. Obesity (BMI ≥ 25 kg/m^2^) was present in 34.0% (n = 64) of the subjects, and 2.7% (n = 5) of the subjects were smokers. We did not find a significant association between smoking status and 8-OHdG levels, potentially owing to the small number of smokers. Higher levels of 8-OHdG were found in older age groups (Table 1). The GMs of the blood Pb and Cd concentrations were 14.42 and 1.99 μg/L, respectively, and the GMs of the urinary Pb and Cd concentrations were 1.12 and 3.51 μg/g Cr, respectively. The correlations between each variable and 8-OHdG as well as the comparisons of the distribution for each variable between the 8-OHdG categories are shown in Table 2. The Spearman’s correlations between 8-OHdG and age, blood Pb concentration, urinary Pb concentration, urinary Cd concentration, vigorous exercise, bicycle riding or walking, and driving an automobile were significant. Among these variables, driving an automobile was negatively associated with 8-OHdG, while the remaining variables were positively associated with 8-OHdG. There were significant differences in blood Pb, urinary Pb, and urinary Cd concentrations between the four 8-OHdG categories, based on the ANOVA test. There were also significant differences in age, bicycle riding or walking, and driving an automobile between the four 8-OHdG categories, based on the Kruskal–Wallis test. Additionally, significant differences in bicycle riding or walking, watching TV or reading, and strenuous housework were observed between the >75th percentile and 51–75th percentile 8-OHdG categories, based on the Mann–Whitney U test. Between the >75th percentile and 26–50th percentile 8-OHdG categories, significant differences were found in urinary Pb concentrations, urinary Cd concentrations, and driving an automobile. Between the >75th percentile and ≤25th percentile 8-OHdG categories, significant differences were found in age, blood Pb concentrations, urinary Cd concentrations, bicycle riding or walking, and driving an automobile (Table 2). The subjects had limited participation in physical activity including moderate exercise, vigorous exercise, resistance training, and carpentry work, resulting in a median of 0 min/week for the four 8-OHdG categories. We did not exclude the ‘Vigorous exercise’ item, which was significantly correlated with 8-OHdG, in the further analysis despite the limited involvement in this type of physical activity, because it was the most interesting observation.

**Table 1:** Characteristics of and 8-hydroxydeoxyguanosine (8-OHdG) levels in female subjects

Variables	8-OHdG (ng/mg Cr)		
	n (%)	Median (min–max)	p^a^
Age (years)			
<50	39 (20.7 %)	3.30 (1.11–14.80)	
50–59	72 (38.3 %)	5.03 (1.48–18.21)	
≥60	77 (41.0 %)	6.56 (1.41–14.41)	0.00^a^
BMI (kg/m^2^)			
<18.5	3 (1.6 %)	8.15 (3.07–9.88)	
18.5–24.9	121 (64.4 %)	5.33 (1.11–14.80)	
≥25	64 (34.0 %)	5.01 (1.48–18.21)	0.73^a^
Smoking status			
Smoker	5 (2.7 %)	5.09 (1.64–14.80)	
Non-smoker	183 (97.3 %)	5.26 (1.11–18.21)	0.76^b^
Alcohol consumption status			
Alcohol consumption	76 (40.4 %)	5.12 (1.41–11.39)	
No alcohol consumption	110 (58.5 %)	5.36 (1.11–18.21)	
Previous alcohol consumption	2 (1.1 %)	4.84 (4.33–5.36)	0.39^a^
Total	188 (100 %)	5.25 (1.11–18.21)	

^a^Median 8-hydroxydeoxyguanosine (8-OHdG) values were compared between age (<50, 50–59, ≥60 years), body mass index (BMI) (<18.5, 18.5–24.9, ≥25 kg/m^2^), and alcohol consumption (alcohol consumption, no alcohol consumption, previous alcohol consumption) groups using Kruskal–Wallis tests

^b^Median 8-OHdG values were compared between the smoking status (smoker and non-smoker) groups using Mann–Whitney tests

**Table 2:** Comparison of variables by quartiles of urinary 8-hydroxydeoxyguanosine (8-OHdG) and correlations of the variables with 8-OHdG levels

Variables	Quartiles of 8-OHdG values						
	≤25th percentile	26–50th percentiles	51–75th percentiles	>75th percentile	Total	p	R^c^
	≤3.68 (n = 47)	3.68–5.25 (n = 47)	5.25–7.47 (n = 47)	>7.47 (n = 47)	(n = 188)		
	Mean (SD), GM (GSD), or median (min–max)	Mean (SD), GM (GSD), or median (min–max)	Mean (SD), GM (GSD), or median (min–max)	Mean (SD), GM (GSD), or median (min–max)	Mean (SD), GM (GSD), or median (min–max)		
Age (years)^f^	50 (39–72)	56 (35–75)	62 (38–77)	63 (47–73)^△^	57 (35–77)	0.00^b^	0.35*
BMI (kg/m^2^)^d^	23.70 (2.72)	24.03 (3.14)	24.18 (2.93)	23.60 (2.97)	23.88 (2.93)	0.75^a^	0.02
Blood lead (μg/L)^e^	13.43 (1.04)	14.02 (1.05)	14.18 (1.04)	16.19 (1.06)^△^	14.42 (1.02)	0.04^a^	0.18*
Urinary lead (μg/g Cr)^e^	1.06 (1.08)	1.01 (1.06)	1.11 (1.05)	1.32 (1.08)^☆^	1.12 (1.04)	0.03^a^	0.20*
Blood cadmium (μg/L)^e^	2.06 (1.08)	1.94 (1.07)	2.09 (1.06)	1.87 (1.06)	1.99 (1.03)	0.58^a^	−0.08
Urinary cadmium (μg/g Cr)^e^	3.27 (1.08)	2.99 (1.09)	3.90 (1.06)	4.00 (1.07)^☆△^	3.51 (1.04)	0.01^a^	0.22*
Physical activity (min/week)^f^							
Voluntary exercise							
Moderate exercise	0 (0–480)	0 (0–900)	0 (0–960)	0 (0–1440)	0 (0–1440)	0.85^b^	0.02
Vigorous exercise	0 (0–120)	0 (0–0)	0 (0–360)	0 (0–630)	0 (0–630)	0.09^b^	0.15*
Resistance training	0 (0–120)	0 (0–0)	0 (0–240)	0 (0–0)	0 (0–240)	0.14^b^	−0.07
Bicycle riding or walking	0 (0–800)	70 (0–500)	0 (0–1800)	120 (0–700)^◇△^	40 (0–1800)	0.01^b^	0.19*
General everyday activities							
Meals	420 (200–1050)	430 (0–1100)	450 (70–1260)	420 (180–1800)	420 (0–1800)	0.72^b^	−0.05
Watching TV or reading	1050 (70–5400)	1050 (150–2940)	840 (70–5400)	1380 (100–7200)^◇^	1050 (70–7200)	0.11^b^	0.12
Driving an automobile	260 (0–4480)	210 (0–3000)	200 (0–1000)	200 (0–5400)^☆△^	210 (0**–**5400)	0.01^b^	−0.24*
Interesting activities at home	1050 (70–3780)	1050 (0–6000)	840 (0–2800)	1200 (0–4200)	1050 (0**–**6000)	0.41^b^	−0.05
Activity at work							
Desk work	60 (0–4100)	300 (0–2940)	0 (0–3960)	140 (0–7200)	60 (0**–**7200)	0.15^b^	−0.05
Strenuous housework	140 (0–1200)	105 (0–840)	60 (0–630)	150 (0–3600)^◇^	120 (0**–**3600)	0.06^b^	0.01
Active work	60 (0–3360)	120 (0–2520)	0 (0–3360)	180 (0–2520)	75 (0**–**3360)	0.83^b^	−0.01
Carpentry work	0 (0–240)	0 (0–540)	0 (0–300)	0 (0–210)	0 (0**–**540)	0.48^b^	−0.10

* p < 0.05 based on the Spearman’s rank correlation analysis

^◇^ p < 0.05 for the comparisons of the mean and GM using Student’s *t* test or the median using Mann–Whitney U tests between the >75th and 51–75th percentile categories

^☆^ p < 0.05 for the comparisons of the mean and GM using Student’s *t* test or the median using Mann–Whitney U tests between the >75th and 26–50th percentile categories

^△^ p < 0.05 for the comparisons of the mean and GM using Student’s *t* test or the median using Mann–Whitney U tests between the >75th and ≤25th percentile categories

^a^Mean body mass index (BMI) and the geometric mean (GM) of the heavy metal exposure variables were compared between the 4 quartile categories of 8-OHdG using ANOVA tests

^b^Median age and physical activity were compared between the 4 quartile groups of 8-OHdG using Kruskal–Wallis tests

^c^Spearman’s rank correlation coefficient between 8-OHdG and each variable

^d^Results shown as mean (standard deviation [SD])

^e^Results shown as GM (geometric standard deviation [GSD])

^f^Results shown as median (min–max)

**Fig. 1:**
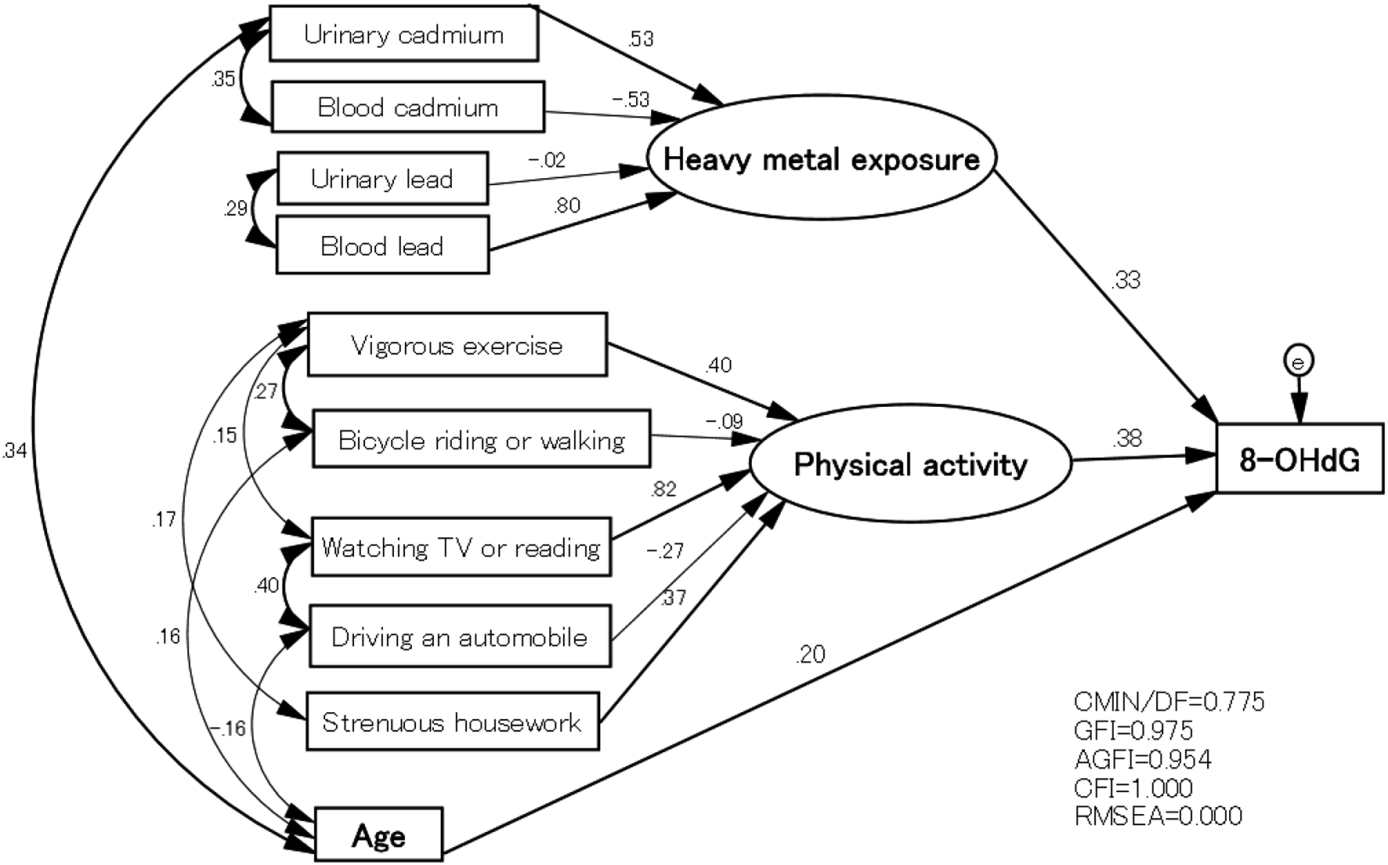
Results of the structural equation modeling (SEM) analysis. The standard β analysis result produced by Amos: the standard β from Heavy metal exposure, Physical activity, and Age for 8-OHdG are 0.33, 0.38, and 0.20, respectively. The model fit test result produced by Amos: CMIN/DF (Chi square/degree of freedom ratio) = 0.775, CMIN/DF ratio values <2 are widely considered to represent a minimally plausible model; CFI (comparative fix index) = 1.000, close to 1 indicates a very good fit; GFI (Goodness of Fit Index) = 0.975, GFI > 0.95 indicates a good fit; AGFI (adjusted GFI) = 0.954, AGFI > 0.9 indicates a good fit; RMSEA (root mean square error of approximation) = 0.000, there is good model fit if RMSEA is ≤0.05

Based on our hypothesis, we created an SEM model using all the heavy metal exposure variables and other variables that were significantly correlated with 8-OHdG or were significantly different between the 8-OHdG categories, as reported in Table 2. Although some of the correlations between these variables and 8-OHdG were poor, the maintenance of these correlations could be determined when the variables were combined with other co-factors in the SEM analysis. Based on the modification indices in AMOS, we deleted some correlation paths if the explanatory variable was unrelated. The final model was good: CMIN/DF = 0.775, CFI = 1.000, GFI = 0.975, AGFI = 0.954, RMSEA = 0.000. The standardized regression coefficients of the variables used in the path models are shown in Table 3. The latent variables of ‘Heavy metal exposure’ and ‘Physical activity’ were positively associated with 8-OHdG levels (β = 0.33 and 0.38, respectively). The observed variable age was positively associated with 8-OHdG levels (β = 0.20). The latent variable of ‘Heavy metal exposure’ was positively associated with urinary Cd concentrations and blood Pb concentrations (β = 0.53 and 0.80, respectively) and negatively associated with blood Cd concentrations (β = −0.53). Urinary lead significant positive associated with 8-OHdG, but lost significance in SEM model. The latent variable of ‘Physical activity’ was positively associated with vigorous exercise, watching TV or reading, and strenuous housework (β = 0.40, 0.82, and 0.37, respectively) in the SEM analysis. Significant positive and negative correlations were present between 8-OHdG and bicycle riding or walking and driving an automobile, respectively, in the correlation analyses; however, the correlations were no longer significant in the final SEM model (Table 3). Additionally, significant correlation coefficient estimates (p < 0.05) were observed in the SEM analysis between the explanatory variables ‘urinary cadmium’ and ‘blood cadmium’ (r = 0.35), ‘urinary lead’ and ‘blood lead’ (r = 0.29), ‘urinary cadmium’ and ‘age’ (r = 0.34), ‘vigorous exercise’ and ‘bicycle riding or walking’ (r = 0.27), ‘vigorous exercise’ and ‘watching TV or reading’ (r = 0.15), ‘vigorous exercise’ and ‘strenuous housework’ (r = 0.17), ‘bicycle riding or walking’ and ‘age’ (r = 0.16), ‘driving an automobile’ and ‘age’ (r = −0.16), ‘watching TV or reading’ and ‘driving an automobile’ (r = 0.40) (Fig. 1).

**Table 3:** Standardized regression coefficients of the variables used in the structural equation modeling to determine associations between heavy metal exposure, physical activity, age and 8-hydroxydeoxyguanosine (8-OHdG) levels (N = 188)

Dependent variable	Independent variable	Standard *β*	p
8-OHdG (measured variable)	Heavy metal exposure	0.33	–^a^
	Physical activity	0.38	–^a^
	Age	0.20	0.00
Heavy metal exposure (latent variable)	Urinary cadmium	0.53	0.01
	Blood cadmium	−0.53	0.01
	Urinary lead	−0.02	0.94
	Blood lead	0.80	0.00
Physical activity (latent variable)	Vigorous exercise	0.40	0.02
	Bicycle riding or walking	−0.09	0.60
	Watching TV or reading	0.82	0.00
	Driving an automobile	−0.27	0.13
	Strenuous housework	0.37	0.02

^a^p values could not be calculated because the independent variable was a latent variable

## Discussion

We hypothesized that heavy metal exposure and other co-factors might be related with oxidative stress. We conducted a study to evaluate 8-OHdG, heavy metal (including Pb and Cd) exposure, physical activity, smoking status, age, and other factors in a regional Japanese female population.

Our research suggests that continuous exposure to heavy metals increases oxidative stress. Urinary Pb, blood Pb, and urinary Cd concentrations were significantly correlated with 8-OHdG concentrations. By including other co-factors in the final SEM analysis, blood Pb concentrations and urinary Cd concentrations were significantly and positively association with the ‘Heavy metal exposure’ variable (*β* = 0.80 and 0.53, respectively), which positively indicated the urinary 8-OHdG concentration. Diagnosis of Pb exposure is mainly based on blood Pb levels, it is the best available marker of current and recent Pb exposure (Lowry 2010), and the adverse health effects have been observed with varying blood Pb levels, from low to high (Perth District Health Unit 2014). In the SEM analysis, urinary Pb concentrations were no longer significant, and blood Pb concentrations were significantly associated with ‘Heavy metal exposure’, which could indicate that blood Pb concentrations have a stronger positive effect for ‘Heavy metal exposure’. A previous study indicated that blood Cd concentrations are mainly influenced by the last few months of exposure and less by the body burden in the general population without occupational exposure (Lauwerys et al. 1994). The subjects in this study did not have occupational exposure, and most of the subjects were non-smokers (97.3 %); therefore, food was likely the major source of Cd exposure. Cd absorption can be affected by many factors such as age, sex, and nutritional status, and higher Cd absorption rates were previously found with younger age in both animal and human studies (Engström and Nordberg 1979; Horiguchi et al. 2004b). In the present study, blood Cd concentrations were not correlated with 8-OHdG and were negatively associated with ‘Heavy metal exposure’ in the SEM analysis, this result could be indirectly affected by other co-factors such as age, which was significantly correlated with 8-OHdG. Further studies are necessary to elucidate these relationships. The urinary Cd concentration is a useful estimate for long-term chronic Cd exposure and total body burden (Järup 2002). In the present study, 8-OHdG and urinary Cd concentrations were significantly correlated. A study among women also found a significant correlation between 8-OHdG levels and urinary Cd concentrations, but not with blood Cd concentrations, indicating that oxidative stress might be affected by long-term Cd retention rather than short-term exposure (Engström et al. 2010). In the SEM analysis, urinary Cd concentrations positively affected the ‘Heavy metal exposure’ variable, even when combined with age and other factors, indicating that urinary Cd concentration is a useful Cd exposure index for oxidative stress, supporting the findings of a previous study in which urinary 8-OHdG concentrations were correlated with urinary Cd concentrations (r = 0.46, p < 0.0001) and with the combined exposure index (Pizzino et al. 2014).

We found that oxidative stress-related 8-OHdG levels increased with age, which confirmed the findings of the study by Muller et al. (2007). Another study in Japanese women found an association between reductions in BMI and elevated urinary 8-OHdG levels (Mizoue et al. 2006). A tendency for higher 8-OHdG levels in subjects with BMIs < 18.5 kg/m^2^ was found in the present study; however, this was not statistically significant because of the small sample size. Tobacco smoking was not significantly associated with 8-OHdG values because most of the female subjects were not smokers. Exercise is associated with increased free radical production and ROS generation, and intensity is a critical factor in exercise-induced oxidative stress (Davies et al. 1982; McBride et al. 1998). In the present study, intense physical activity such as vigorous exercise (standard *β* = 0.40) and strenuous housework (standard *β* = 0.37), which positively indicated ‘Physical activity,’ also positively indicated the 8-OHdG concentrations. Sedentary behavior, which includes TV viewing and reading, has been identified as an independent risk factor for several diseases (Wilmot et al. 2012). Previous studies suggested that sedentary lifestyle is a high-risk behavior which might result in low shear stress, leading to ROS generation and increased oxidative stress (Szostak and Laurant 2011; Takahashi et al. 2015; Thosar et al. 2012). In the present study, we found that the sedentary behavior of ‘watching TV or reading’ positively indicated ‘Physical activity,’ which also positively indicated the 8-OHdG concentrations (standard *β* = 0.82), supporting the findings of the previous studies. Bicycle riding or walking and driving an automobile were significantly associated with 8-OHdG in the correlation analyses, but not in the final SEM analysis. It is noteworthy that significant positive or negative correlations were present between these two variables and the co-factor ‘age’ in the SEM analysis. Therefore, the association between these variables and 8-OHdG were more convincing in the SEM models, when they were combined with other confounding factors.

Furthermore, 8-OHdG has been analyzed using both high performance liquid chromatography (HPLC) and enzyme-linked immunosorbent assay (ELISA) methods (Tamae et al. 2009; Witherell et al. 1998). The mean 8-OHdG concentration in the present study (5.78 ± 2.91 ng/mg Cr) was lower than that determined previously using ELISA (Saito et al. 2013; Shimoi et al. 2002; Witherell et al. 1998). The limitation of this study is that the region is in an agricultural area of Japan, most of the subjects were farmers or housewives, and the subjects spent more time being physically active during their general everyday activities at home and doing strenuous housework than during voluntary exercise and doing carpentry work.

## Conclusion

In summary, we hypothesized that heavy metal exposure and the concurrent presence of lifestyle factors and age are related with oxidative stress. The most positive associations with 8-OHdG were present for several items of heavy metal exposure (urinary Cd concentrations and blood Pb concentrations), physical activity (vigorous exercise, watching TV or reading, and strenuous housework), and age. The findings of this study suggest that oxidative stress is related with exposure to heavy metals such as Pb and Cd, and physical activity in combination with aging. It is important to reduce chronic heavy metal exposure and manage lifestyle factors during aging to prevent the adverse health effects caused by oxidative stress. Further studies with other female populations with different lifestyles and heavy metal exposure levels are required to confirm and strengthen our findings.
